# Recent Development in Plasmonic Nanobiosensors for Viral DNA/RNA Biomarkers

**DOI:** 10.3390/bios12121121

**Published:** 2022-12-03

**Authors:** Dong Hyeok Park, Min Yu Choi, Jin-Ha Choi

**Affiliations:** School of Chemical Engineering, Clean Energy Research Center, Jeonbuk National University, Jeonju 54896, Republic of Korea

**Keywords:** plasmonic nanomaterials, viral DNA, viral RNA, early diagnosis, nanobiosensors

## Abstract

Recently, due to the coronavirus pandemic, the need for early diagnosis of infectious diseases, including viruses, is emerging. Though early diagnosis is essential to prevent infection and progression to severe illness, there are few technologies that accurately measure low concentrations of biomarkers. Plasmonic nanomaterials are attracting materials that can effectively amplify various signals, including fluorescence, Raman, and other optical and electromagnetic output. In this review, we introduce recently developed plasmonic nanobiosensors for measuring viral DNA/RNA as potential biomarkers of viral diseases. In addition, we discuss the future perspective of plasmonic nanobiosensors for DNA/RNA detection. This review is expected to help the early diagnosis and pathological interpretation of viruses and other diseases.

## 1. Introduction

There are an estimated nonillions (10^31^) individual viruses in the world, and more than 7000 viral genotypes have been extensively studied [1]. Viruses are non-cellular structured infectious pathogens that cannot reproduce outside of host cells and are infectious agents that can infect all cellular organisms (prokaryotic, eukaryotic, and archaea). Depending on the genetic material (DNA or RNA), it is classified as a DNA virus (herpes viruses, smallpox viruses, adenoviruses, human papillomavirus, para retroviruses, etc.) [2], RNA virus (Ebola, hepatitis C, influenza, severe acute respiratory syndrome (SARS), and poliomyelitis, etc.) [3]. Early diagnosis of these viral diseases is essential to prevent the spread of infection and increase survival rates. Recently, with high sensitivity and specificity, polymerase chain reaction (PCR) techniques can amplify certain virus genes to identify small amounts of viruses, but this method has limitations in sampling errors or insufficient viral load (false negatives) [4]. In addition, it requires many reagents (nucleotide mix, target DNA primers, reverse transcriptase, etc.), specialized experimental equipment, and skilled technicians to perform the analysis, which is time-consuming and expensive [5]. In order to overcome these limitations and improve the sensitivity of biosensors, various studies using nanomaterials are being conducted (electrochemiluminescent biosensor [6], fluorescence [7], plasmon-enhanced fluorescence [8], SERS biosensor [9,10], colorimetry biosensor [11], etc.). Nanomaterials have high reactivity of their large surface area and depending on the type or characteristics, they have high thermal and electrical conductivity [12], superior mechanical properties [13], excellent support for catalysts [14], and antimicrobial activity [15]. Among the various nanomaterials, plasmonic nanomaterials can dramatically improve sensor sensitivity through signal amplification. A plasmon is a quasiparticle in which free electrons in a metal vibrate collectively, and surface plasmon is the collective vibration of an electron cloud on a metal surface excited by incident electromagnetic radiation and interacting strongly with light [16]. Irradiating the light on metal nanoparticles at their plasmon frequency produces strong electric fields at the nanoparticle surface [17,18]. The frequency of this resonance depends on the nanoparticle size, shape, material, and surrounding environment [16]. For example, it is possible to shift the plasmon resonance of gold from the visible range into the infrared wavelength range by minimizing the nanoparticle size [19]. The strong electric fields generated near plasmon resonance metal nanoparticles are used as enhancement factors in surface-enhanced Raman spectroscopy (SERS) [20]. In this review, we introduce sensitive viral DNA/RNA measurements using the plasmonic effect of nanomaterials. Moreover, we explain CRISPR-based plasmonic nucleic acid biosensors that have been drawing attention recently and discuss prospects.

## 2. Signal Amplification Strategies Using Plasmonic Nanomaterials on Nanobiosensors

The unique properties of plasmonic nanomaterials have been frequently applied to the development of superior biosensors for the improvement of biosensor performance, such as the sensitive detection of biomarkers [21,22,23,24,25,26,27,28]. Surface plasmon resonance (SPR) is the resonant coupling of electromagnetic waves to collective oscillations of free electrons on the surface of plasmonic materials, including gold (Au) and silver (Ag) [29,30,31]. Through this resonant coupling phenomenon, which occurs at the interface between dielectric and plasmonic materials, the incident light can be enhanced and confined. These properties make plasmonic nanomaterials suitable for exceptional diagnostic platforms, owing to their high sensitivity to changes in dielectric properties at the surface of plasmonic nanomaterials. Among several plasmonic nanomaterials, Au and Ag are the most extensively studied to develop molecular diagnostics platforms. This phenomenon of coupling resonant incident light to the collective oscillation of the electrons on the surface of the noble nanomaterials leads to its enhanced or altered optical absorption. The plasmonic effect induced enhancing and altering optical absorption, and a strong scattering effect when the particle size is above a few tens of nanometers. The extent of the enhancing, altering, and scattering efficiency adjusts depending on the size, shape, and composition of the plasmonic nanomaterials.

In biosensing applications, the interaction between biomolecules and plasmonic materials changes various optical properties of plasmonic materials (e.g., plasmonic resonance wavelength, the range of absorbance values, etc.). By measuring the changed optical properties, it is possible to detect a biological material, and this change occurs sensitively. For example, the intrinsic emission intensity of a fluorescent material decreases (quenching effect) at a constant distance from the plasmonic nanoparticles, changes in wavelength (FRET), and is amplified (MEF) [32,33,34]. In addition, plasmonic nanostructures developed as electromagnetic fields were enhanced relative to the incident light. The magnitude of the enhancement depends on the degree of confined nanostructure for the enhanced electromagnetic fields. For example, nanostructures with sharp edges and a vertex could provide a hot spot, which creates very large, highly localized field enhancements. These field enhancements can be induced by surface-enhanced Raman scattering (SERS), which is inelastic scattering from molecular vibrational modes [35,36,37,38]. There have been several attempts to sensitively measure biomarkers for early detection of fetal diseases, including viral nucleic acids, by using these plasmonic properties. In the next chapters, we will examine a representative case of the recently published plasmonic nanobiosensor for measuring viral nucleic acids.

## 3. Sensitive Plasmonic Nanobiosensors for Viral DNA Detection

Most DNA and RNA viruses show similar structures, such as the capsid proteins and nucleic acids, including double- or single-stranded DNA or RNA [39,40]. As mentioned, the typical detection methods for viral DNA and RNA are nucleic acid amplification-based approaches, such as PCR and LAMP, which require multiple stepwise temperature cycles [41,42,43,44]. In the case of general PCR, the amplified nucleic acid was converted to visualized materials by gel electrophoresis. Furthermore, real-time PCR techniques have been utilized to confirm the real-time amplification of nucleic acids. In both methods, fluorescent molecules are commonly used as probe material. Fluorescent molecules have the advantage that they can be easily identified with the naked eye using a simple light source and can be quantified according to fluorescence intensity. Using these methods, quantitative results can be achieved, resulting in precise and early diagnosis. However, nucleic acid amplification-based methods need improvement due to the experimental process being complicated, and accordingly, it is cost- and time-ineffective. The fluorescence-based measurement method has the advantage that the fluorescence signal can be amplified using various plasmonic nanomaterials, and many attempts have been made to measure viral nucleic acids (Table 1).

### 3.1. FRET

FRET is a distance-dependent energy-transfer phenomenon between the donor (fluorophore) and acceptor (another fluorophore or quencher) [45,46,47]. FRET could show when the distance between the donor and acceptor is below 10 nm. If the distance is above 10 nm, the efficiency of FRET is drastically decreased, and the emission intensity of the donor fluorophore is recovered. Therefore, the distance between the donor and acceptor is a key factor in determining the FRET phenomenon. When the binding phenomenon of viral DNA changes the ‘distance’, the fluorescence signal changes, and DNA quantification is possible using this phenomenon. Based on this principle, FRET is used for the detection and quantification of nucleic acids extracted from body fluid, such as blood, urine, sweat, etc. However, because the concentrations of nucleic acids are very low, a highly sensitive detection method is essential for the precise and early diagnosis of virus infection. A variety of nanomaterials, including plasmonic nanoparticles, can act as donors and acceptors that cause FRET, which makes target nucleic acid measurements more sensitive. There are lots of studies on FRET-based biosensors for viral DNA detection. Zeng et al. developed the FRET probe, which consists of a One-Donor-Four-Acceptors (D–4A) assembly, for sensitive detection of the Hepatitis C virus (HCV) DNA [48]. Most of the FRET probes showed inefficient energy transfer due to the weak dipole–dipole interactions between donor and acceptor. To overcome this limitation, a multi-acceptor was applied to one donor in this biosensing system. In detail, the D–4A FRET probe was assembled by separating three single-stranded DNA (ssDNA) strands, two were target-specific nucleic acid sequences with acceptors (BHQ-1) at both 5′- and 3′- ends. The other sequence is functionalized by the FAM. When HCV DNA was bound to the two specific nucleic acids, fluorescence intensity was drastically increased because four acceptors were far away from the FAM. This system can significantly reduce the background signal and improve the detection sensitivity to as low as 24.57 nmol/L. Vafaei et al. developed a micellar nanostructure-based DNA sensing system composed of lipid and two nucleic acid probes with a donor and acceptor [49]. Before the target DNA-binding reaction, two complimentary DNA strands with Cy3 and Cy5 were encapsulated into each two micelle structures. When the target DNA was bound to the two DNAs, two different micelles could fuse with each other, and the distance between the donor and acceptor became shortened. Thus, mixed micelles showed the FRET phenomenon, whose signal intensity was greater than the conventional single FRET reaction. In the case of the limits of detection (LOD), the mixed micelle system was improved by factors of <20, and the FRET efficiency was enhanced by three times. As such, in order to improve the sensitivity, the FRET signal was improved by increasing the amount of donor and acceptor corresponding to the target DNA. These methods are useful to improve the sensitivity, but on the other hand, there is a limit in increasing the amount of donor and acceptor, so there is a limit in measuring very low concentrations of viral DNA.

**Figure 1:**
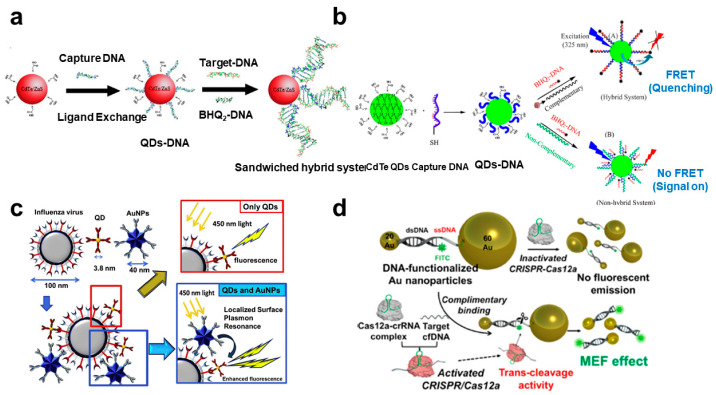
Viral DNA-target fluorescence biosensors using plasmonic nanomaterials: (**a**) Schematic diagram of the QDs-DNA-based target DNA detection system. This figure is reproduced from [48] (© 2022 Elsevier B.V.); (**b**) Schematic diagram of the FRET-based SARS-CoV2-DNA detection. This figure is reproduced from [49] (© 2022 Royal Society Chemistry); (**c**) Plasmonic AuNPs enhanced fluorescence signal intensity of QD through the sandwich structure with the influenza virus. This figure is reproduced from [50] (© 2022 Elsevier B.V.); (**d**) MEF-based DNA detection system using plasmonic Au-assisted MEF effect by CRISPR-Cas12a reaction. This figure is reproduced from [52] (© 2022 American Chemical Society).

As described above, plasmonic nanomaterials have great optical properties, originating from the surface plasmon resonance. In the case of FRET, Au nanoparticle is one of the good acceptors and can absorb emitting light from the fluorescent materials (donor) and hinder the emission. Lu et al. developed the Au nanorod-based FRET biosensor for the detection of the hepatitis B (HBV) virus with a simple strategy [50] (Figure 1a). FAM-labeled complementary ssDNA could be attached to the surface of the Au nanorod due to the electrostatic interaction. On the other hand, dsDNA could be more attached to the Au because of the stronger negative charge of the target DNA-complimentary DNA complex than the ssDNA. Using this simple schematic, the high level of target DNA induced a strong FRET effect. The dynamic range of the HBV DNA is 0.045 to 6.0 nM, with 15 pM LOD. Liu et al. exhibited the upconversion nanoparticle (UCNP)-based photostable FRET biosensing system for long-chain HIV DNA detection [51]. A typical FRET biosensing system could not detect long-chain DNA because a few nanometers distance is essential to induce the FRET effect. To solve this problem, a head-to-tail structure was applied to generate FRET between Au nanoparticle (AuNP) and UCNP. Unlike the head-to-head structure, the head-to-tail sandwich hybridization structure can make the distance between the AuNP and the UCNP close regardless of the length of the target DNA. In addition, AuNP and UCNP showed excellent photostable properties, compared to organic fluorescent dyes. Therefore, the 52 bp HIV DNA target was successfully measured by using this stable system with a nanomolar detection limit. Bardajee et al. presented a similar sandwich hybrid system using QD and BHQ-DNA for the detection of the SARS-CoV-2 genome (Figure 1a) [52]. The strong point of these FRET-based sensors is that they could be applied to on-site and real-time detection using real samples, such as saliva. They also showed a fluorescence CdTe quantum dots-DNA (QDs-DNA) nanosensor for efficient detection of SARS-CoV-2 viral DNA and RNA using the FRET effect via forming a sandwich structure [53] (Figure 1b). Thiolated capture DNA was immobilized on the surface of QD, and BHQ-DNA could be complimentary bound to the capture DNA. If SARS-CoV-2 viral DNA or RNA was introduced in this system, viral DNA bound to the capture DNA competitively with BHQ-DNA, and accordingly, as the concentration of the target DNA increases, the FRET phenomenon was inhibited, and it could be confirmed that the intensity of fluorescence emission of the QD was increased. The partial DNA of the SARS-CoV-2 genome was simply and successfully measured with a 2.52 nM LOD.

### 3.2. MEF

Besides the FRET effect, the combination of plasmonic nanomaterials and fluorescence molecules can induce a change in fluorescence property, which is an enhanced emission intensity [34]. The surface plasmon properties of plasmonic materials convert from optical energy to fluorescence emission of adjacent fluorescence materials. Proximal fluorescence molecules can be induced MEF, which significantly enhances the intensity of the fluorescent signal. Using this converting and enhancing effect could improve the sensitivity of biomarker detection, including virus-related materials. Takemura et al. reported on the highly sensitive detection of influenza virus H1N1 using a sandwich immunoassay strategy based on a plasmonic AuNP and CdSeTeS QD [8] (Figure 1c). The mechanism of the biosensor was an immunoassay between the surface antigen of H1N1 and antibody functionalized AuNPs. If another specific antibody functionalized CdSeTeS QD was bound to the H1N1 surface, the emission intensity of QDs could be enhanced due to the plasmonic effect of adjacent AuNPs. The LOD for H1N1 was 0.03 pg/mL in deionized water and 0.4 pg/mL in human serum, respectively. Nasrin et al. developed influenza virus-detectable fluorescence biosensors by using CdZnSeS/ZnSeS QD and AuNP, conjugated with peptide sequence [54]. In this system, the distance between QD and AuNP was optimal to amplify the fluorescence signal by peptide sequence. However, the fluorescent emission of QD could be quenched due to the hindrance of the influenza virus, which was bound to the specific antibody at the center of the peptide. Thus, the influenza virus could be detected by reducing the fluorescence intensity of the QD. The detection limit was 17.02 fg/mL, ranging from 10^−14^ to 10^−9^ g/mL. Choi et al. demonstrated the CRISPR-Cas12a-based DNA biosensors by integrating the MEF effect for signal amplification and sensitive detection [5] (Figure 1d). Typical CRISPR-based sensing system integrated nucleic acid amplification step for sensitive and effective detection. However, the target amplification strategy involves complex steps, high costs, and time-consuming issues. MEF could be one of the potential candidates to overcome this limitation. In this study, DNA could be measured by ten femtomolar levels without a target amplification step. Wei et al. showed the digitalizing biosensor for the rapid detection of viral DNA using loop-mediated isothermal amplification (LAMP) and Au nanoarray [55]. The rough Au sensing surface could provide the hot spot to generate the MEF. For the detection of hepatitis virus DNA, Bst polymerase was functionalized on the Au nanopattern. The plasmonic property of Au and LAMP-mediated target DNA amplification could induce the sensitive detection of DNA by digitized counting of amplification of a fluorescent signal. The detection limit of this MEF-LAMP system was 4 pg/μL, which was 10 times better than the conventional LAMP system. Jin et al. exhibited the sensitive HBV DNA detection platform using silver nanoparticle (AgNP) aggregates. AgNPs were functionalized to the capture DNA, and they could be aggregated by HBV DNA [56]. Target DNA could induce the increment of the fluorophore with capture DNA, and MEF effect by aggregated AuNPs. This AgNP-based MEF sensing system achieved a 50 fM LOD, ranging from 100 fM to 10 nM. In addition, single-mismatched DNA could be distinguished from the target HBV DNA. Similar to the FRET biosensing methods, MEF-based viral DNA sensors could apply to real-time and in situ diagnosis with improved sensitivity.

**Table 1:** Comparison of fluorescent-based plasmonic nanobiosensors for viral DNA detection.

Analytical Method	Feature	Target	Required Time	Detection Limit	Ref
FRET	One-donor-four-acceptors FRET probe for the HCV DNA detection	HCV DNA	40 min	24.51 nM	[48]
FRET	Lipid oligonucleotide FRET probes incorporated into micellar scaffolds	cDNA	30 min	0.625 nM	[49]
FRET	FAM-ssDNA–CTAB–AuNRs ternary complex	HBV DNA	50 min	15 pM	[50]
FRET	NaYF4:Yb,Er nanoparticles with carboxylic acid groups and report DNA-modified AuNP	HIV DNA	20 min	3 nM	[51]
FRET	DNA-Conjugated CdTe Quantum Dots Nanoprobe	SARS-CoV-2 genome	30 min	2.52 nM	[52,53]
MEF	Anti-neuraminidase (NA) antibody (anti-NA Ab) to thiolated AuNPs and the anti-hemagglutinin (HA) antibody (anti-HA Ab) to alloyed quaternary L-cysteine-capped CdSeTeS QDs	Influenza virus	3 min	0.03 pg/mL	[8]
MEF	Fluorescent CdZnSeS/ZnSeS QDs and AuNPs with target-binding peptide chain	Influenza virus	3 min	17.02 pg/mL	[54]
MEF	DNA-functionalized AuNP pair for CRISPR-Cas12a-based detection	cfDNA (BRCA-1)	30 min	100 fM	[5]
MEF	Isothermal amplification on plasmonic enhanced digitizing biosensor	HBV and HCV DNA	10 min	4 ng/mL	[55]
MEF	AgNPs functioned with recognition probes (Cy3-probe) and hybrid probes	HBV DNA	15 min	50 fM	[56]

### 3.3. Raman

**Figure 2:**
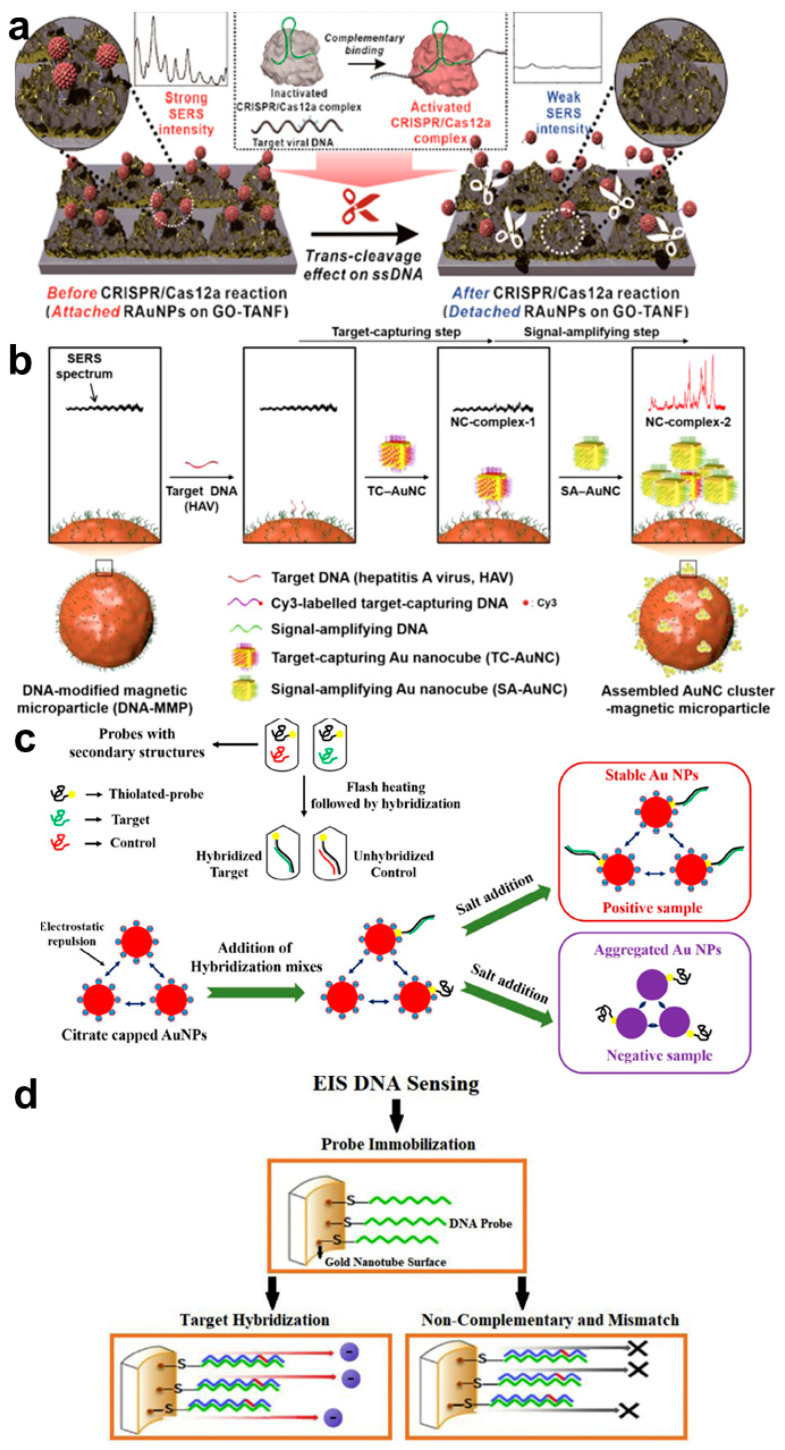
Viral DNA-target SERS and electrochemical biosensors using plasmonic nanomaterials: (**a**) Schematic diagram of the SERS-active viral DNAs detection platform using rough-faced Au nanostructure with GO and Raman dye-functionalized AuNPs. This figure is reproduced from [55] (© 2022 American Chemical Society); (**b**) Schematics of the SERS-based sensitive DNA detection using hierarchic interfacial Au nanocube assembly. This figure is reproduced from [58] (© 2022 American Chemical Society); (**c**) Plasmonic AuNPs-assisted colorimetric biosensor for detection of HCV DNA. This figure is reproduced from [64] (© 2022 Springer); (**d**) Nanoporous Au nanotube-based electrochemical impedance spectroscopy (EIS) biosensors. This figure is reproduced from [65] (© 2022 Elsevier B.V.)

Among the several optical properties, Raman scattering is one of the specific properties of every molecule. Raman is a light scattering phenomenon by incident light. Most of the scattered light from molecules can divide into two scatters, Rayleigh Scatter and Raman scatter, a small amount of light at different wavelengths [57]. This wavelength is decided depending on the chemical structure of the molecules. Thus, Raman spectroscopy could be a formidable analytical method to identify numerous biomarkers. For the diagnosis of virus infection, Raman spectroscopy could be applied to specific detection (Table 2). Tong et al. exhibited the precise detection of HBV infection using 500 patient samples [58]. By integrating the principal component analysis (PCA) and a support vector machine (SVM), the authors developed the modeling and prediction system for the selection of infected samples using the Raman spectra. Using this system showed great accuracy, sensitivity, and specificity of the HBV (93.1%, 100%, and 88%, respectively). However, even though the Raman spectrum provides specific scattering information, signal intensity is naturally too weak to identify viral DNA. To solve this limitation, surface-enhanced Raman scattering (SERS), the Raman signal-amplification method with surface plasmon effects on the surface of noble metal, have been utilized to detect low concentrations of biomolecules, including viral DNA. Choi et al. developed the ultrasensitive viral DNA sensing platform using a rough-faced Au nanostructure and DNA-functionalized graphene oxide (GO) [59] (Figure 2a). For maximizing Raman signal amplification, the plasmonic surface was fabricated with the Ag etching method from the Au–Ag surface to provide lots of hot spots, which induced strong electromagnetic (EM) fields to increase the Raman vibrational signals. In addition, GO was functionalized on the Au surface for the induction of chemical enhancement (CM) of Raman dye-functionalized AuNPs, which was connected by the single-stranded DNA on the GO–Au nanostructured surface. Using this SERS-optimizing system improved the sensitivity of the viral DNAs, including HBV, human papillomavirus 16 (HPV-16), and HPV-18, with a 1 aM LOD. Su et al. showed a similar strategy to detect HPV DNA using a pair of AuNP, which are connected by ssDNA [60]. Raman dye-functionalized two AuNPs modified each capture DNA. The linker DNA was bound to the capture DNAs, forming a sandwich structure, and a pair of AuNP could provide a hot spot for the SERS. If the HPV DNA made a Cas12a-crRNA-DNA complex, the intact linker DNAs were degraded, and the AuNPs were well-dispersed. This effectively prevented the aggregation of AuNPs, and the SERS effect could be decreased. This SERS-based detection method achieves the picomolar LOD of HPV DNA genes within 40 min. Lin et al. fabricated the Au-Os-CO (osmium carbonyl)-Au functionalized SERS active nanostructure for the sensitive detection of cell-free DNA (cfDNA) from Epstein-Barr Virus [61]. The SERS active substrate consisted of a rhenium carbonyl (Re−CO), an Os−CO as an internal reference, and a streptavidin layer to serve as a capture DNA. For the SERS effect, the Au layer was applied to the Os-CO, and daunorubicin (DNR) was presented between Os-CO and Re-CO. Due to the spectral overlap of Os-CO (2113 cm^−1^) and Re-CO (2025 cm^−1^) by the intercalation of DNR, complementary binding of cfDNA could induce an increase in the intensity of 2113/2025 cm^−1^ ratio. The detection range was from 600 to 60,000 copies/mL using an isolated DNA sample from patients and healthy donors. Kim et al. developed the novel hierarchic interfacial Au nanocube for an effective SERS generation [62] (Figure 2b). Because the induction of a strong SERS signal is decided by the gap formation of the plasmonic nanoparticle, two Au nanocubes were (target-capturing and signal-amplifying Au nanocube) utilized to enhance the SERS signal. The Au nanocube complexes on the surface of the magnetic particle could generate a strong SERS signal, corresponding to the hepatitis A virus DNA. Based on this method, Au nanocube-assisted assay makes available for detecting target viral DNA, ranging from 100 aM to 10 pM. Yin et al. exhibited the magnetic field-assisted SERS platform for the ultrasensitive detection of the N gene of SARS-CoV-2 [63]. For the induction of an intense SERS signal, the distance between the Raman probe and plasmonic nanomaterial is one of the critical factors. In the case of DNA detection, the distance is relatively long due to the length of the capture and target DNA. In this study, the magnetic force could decrease the distance and the SERS signal could be more enhanced for the reduction in the coupling distance between the AuNP and reduced GO (rGO). The authors claimed that the SERS signal was 10-fold enhanced compared with the non-magnetic platform and the LOD was around 100 aM. These plasmonic-based SERS analytical methods are effective to improve the detection sensitivity of viral DNA.

### 3.4. Localized SPR (LSPR) and Electrochemical Analysis

Due to the plasmonic effect of the noble metal nanomaterials, they have particular optical properties, such as FRET, MEF, and SERS. Absorbance at a specific wavelength is one of the distinguished phenomena which can be easily observed by the naked eye. In the case of AuNP, the absorbance property depends on the size and the degree of aggregation. If the distance of each AuNP is very short (a few nanometers), the individual localized plasmon fields are coupling, and the absorbance peak moves from blue to red, which shows a change of color [64,65,66] (Table 2). Li et al. demonstrated the viral DNA detection method using capture DNA-functionalized AuNPs, which could be closely presented by the target binding effect [67]. Once viral DNA activated to the nuclease effect of CRISPR-Cas12a, the target DNA was degraded, and the two AuNPs could not bind to each other (no change). The advantages of these color change analyses are visual, a colorimetric readout for deployable to the fields. Prakash et al. developed a sensitive HCV DNA detection system consisting of simple negatively charged AuNPs, which were functionalized by complementary DNA to the HCV DNA [68] (Figure 2c). In this color change sensor, optimal hybridization condition was identified by applying flash heating at 95 °C with citrate buffer. Because the DNA hybridization with viral DNA and thiolated probe DNA could prevent the aggregation of negatively charged AuNP in citrate buffer, the color change reflected the negative viral DNA.

Besides the optical detection strategies for viral DNA sensing, electrochemical methods have been developed using plasmonic nanomaterials due to their great electrical property. Shariati et al. exhibited the impedimetric electrochemical biosensor for the ultrasensitive detection of the human papillomavirus (HPV) DNA [69] (Figure 2d). Using the polycarbonate rod nanopattern, the Au nanotube array was fabricated by electrodeposition and etching methods. Because the surface of the Au nanotube improved the electric field, the HPV-16 DNA could be measured in a sensitive and selective way. The developed electrochemical impedance spectroscopy (EIS)-based biosensor showed the very sensitive detection of DNA, as low as 1 fM, in the linear response ranges of 0.01 pM to 1 μM. Faria et al. developed the Au-coated polyethylene terephthalate (PET) electrode for the taction of Zika and Dengue genome DNA [70]. This disposable and flexible Ayu electrode provided feasible chemical modification of the thiolated capture DNA. This work also used EIS analysis for viral DNA detection and showed 25.0 ± 1.7 nM detection limit with a 1.5 h response time. The advantage of both works was label-free detection, which reduced complicated steps and enabled point-of-care diagnosis. Other work measured Evola DNA using a similar detection strategy [71]. In this work, an alkaline phosphatase enzyme was applied to the electrode for the redox enzyme reaction. The LOD of the Evola virus DNA was 4.7 nM using a standard deviation of a blank sample. Redox reaction could improve the sensitive detection of viral DNA; however, the enzyme should be labeled after the target binding step.

**Table 2:** Comparison of SERS and LSPR-based nanobiosensors for viral DNA detection.

Analytical Method	Feature	Target	Required Time	Detection Limit	Ref
Raman	Raman spectroscopy combined with the airPLS-PCA-PSOSVM model	HBV DNA	-	-	[58]
SERS	Raman-sensitive system composed of ssDNA-immobilized Raman probe-functionalized Au nanoparticles (RAuNPs) on the graphene oxide (GO)/triangle Aunanoflower array.	HBV, HPV-16, HPV-18 DNA	20 min	1 aM	[59]
SERS	AuNPs aggregation-based surface-enhanced Raman scattering (CRISPR/Cas-SERS) platform	HPV DNA	40 min	6.72 pM	[60]
SERS	Au−Os−CO−Au Functionalized SERS-Active Substrate	Epstein-Barr Virus DNA	30 min	600 copies/mL	[61]
SERS	Hierarchic-nanocube-assembly based SERS (H-Cube-SERS) bioassay to controllably amplify the electromagnetic field between gold nanocubes	HAV DNA	30 min	100 aM	[62]
LSPR	Plasmonic CRISPR Cas12a-based assay using DNA-functionalized AuNP	Red-Blotch Viral DNA	15 min	200 pM	[67]
LSPR	Plasmonic gold nanoparticles for ratiometric genosensing of Hepatitis C virus using citrate buffer and flash heating in enhancing the sensitivity	HCV DNA	20 min	-	[68]

## 4. Sensitive Plasmonic Nanobiosensors for Viral RNA Detection

As described in Section 3, viral RNAs have also been detected in a similar way to DNA. In order to detect viral RNA through the PCR or other nucleic acid-based methods, the converting process to cDNA through the reverse transcription method should be added. Therefore, since it is slightly more complicated compared to DNA detection, it is important to simplify the detection method by maintaining or improving measurement sensitivity. Therefore, studies on sensitive and simple detection of viral RNA using the characteristics of plasmonic nanomaterials (MEF, SERS, LSPR, etc.) are also being actively conducted.

### 4.1. Fluorescence Analysis

**Figure 3:**
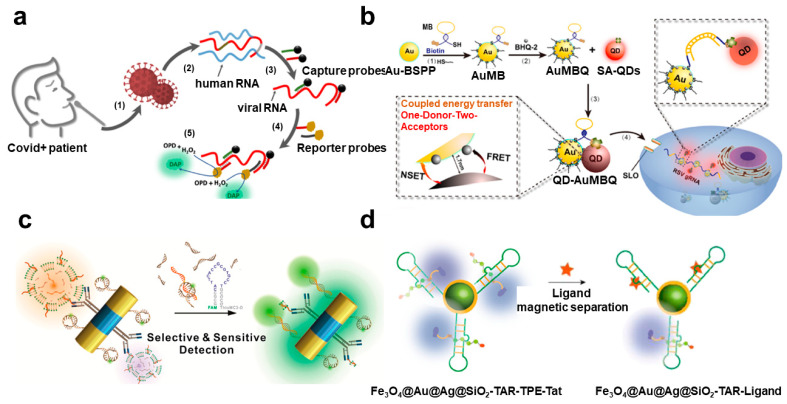
Viral RNA-target fluorescence biosensors using plasmonic nanomaterials: (**a**) Schematic diagram of the magnetofluorescent SARS-CoV-2 biosensor using enzyme-based fluorescence measurement. This figure is reproduced from [68] (© 2022 American Chemical Society); (**b**) One-donor-two-acceptors FRET biosensing system for the gene replication measurement in a living cell. This figure is reproduced from [71] (© 2022 Elsevier B.V.); (**c**) Magnetoplasmonic detection of exosomal RNA based on MEF. This figure is reproduced from [72] (© 2022 American Chemical Society); (**d**) HIV RNA-binding ligand detection system by using plasmonic enhanced fluorescence effect. This figure is reproduced from [74] (© 2022 American Chemical Society.).

For the diagnosis of the viral infection, RNA is also a potential biomarker, similar to the viral DNA. Representatively, the common cold, influenza, and SARS-CoV-2 have RNA as a genetic material to generate a viral disease. Similar to viral DNA assays, RNA is mainly measured using nucleic acid amplification methods such as PCR or using complementary binding phenomenon. Several kinds of research on improving the performance of sensors using plasmonic nanomaterials have been actively conducted (Table 3). In particular, due to the recent coronavirus epidemic, there have been many studies to measure viral RNA. Zayani et al. reported that the SARS-CoV-2 RNA was measured from the positive patient samples using the magnetofluorescence effect [72] (Figure 3a). Magnetic nanoparticle and HRP conjugated DNA probes could hybridize to the viral RNA and the separated by the magnetic force. Conjugated HRP provided the fluorescent signal by oxidization of fluorogenic o-phenylenediamine (OPD) to fluorescent 2, 3-diaminophenazine (DAP). Thus, quantification of the viral RNA was possible to measure the fluorescent signal from DAP. This biosensing system could detect as low as 1000 copies/μL within 30 min. Blumenfeld et al. exhibited an improvement in the performance of the quantitative RT-PCR by using a Au nanorod [73]. The plasmonic effect of the Au nanorod could convert the light into thermal energy. Using infrared (IR) LED and cooling fan, fast thermal cycling was induced to low-cost and time-saving RT-PCR. The suggested PCR-based system could rapidly detect SARS-CoV-2 RNA from a real sample with 2.2–4.4 copies/μL LOD within 30 min. In addition, multiplex detection could be possible by using different fluorescent dyes in the sample of the RT-PCR. Cheong et al. showed a similar plasmonic RT-PCR biosensing system to measure SARS-CoV-2 RNA. It could also rapidly control the temperature by the photothermal conversion of magneto-plasmonic nanoparticles [74]. In addition, these magneto-plasmonic nanoparticles prevented the plasmonic quenching effect by the separation of magneto-plasmonic nanoparticles after the nucleic amplification step. Using this strategy, SARS-CoV-2 RNA can be detected in 17 min with a portable size thermocycler. The LOD was similar to the conventional benchtop PCR equipment, 3.2 copies/μL. Zheng et al. developed the intracellular viral RNA detection platform using a FRET-based system [75] (Figure 3b). In the case of intracellular analysis, reducing the background signal is critical for the improvement of sensitivity because the many intra- and extracellular components could prevent the measurement of fluorescent signals. In this work, a one-donor-two-acceptor system consisting of QD as a donor and BHQ-2, and AuNP as acceptors was applied to inhibit the negative signal of the FRET. This one-donor-two-acceptor nanoprobe showed strong photostability and improvement of signal-to-background ratio. It could be possible to measure spatiotemporal distributed genomic RNA gRNA, which replicated human respiratory syncytial virus (RSV) in live cells. This stable and high energy transfer effect of the nanoprobe could enable monitoring for the long-term in a wide range of virus inoculation doses in real-time. Including the FRET effect, fluorescence analytic methods could be applied to wide diagnostic fields, such as point-of-care, sensitive evaluation, and intracellular analysis.

For the sensitive detection of RNA strands, MEF-based fluorescence biosensors have been developed by using plasmonic phenomena of diverse nanomaterials. Lee et al. fabricated the magneto-plasmonic multifunctional nanorod for the ultrasensitive measurement of exosomal microRNA (miRNA) [76] (Figure 3c). The magnetic property of the particle was utilized for the separation of the concentration of exosome from the stem cell. On the other hand, Au components amplified the fluorescence signal from the molecular beacon, complimentary hybridized with target miRNA. These two functions could provide the sensitive detection platform of the miRNA related to stem cell differentiation. Woo et al. reported the highly sensitive on-site viral RNA detection platform [77]. For the sensitive detection, reverse polymerase amplification (RPA) was conducted for the target amplification on the three-dimensional (3D) Au plasmonic nanoarray composed of Au nanopillar and spherical AuNPs. This 3D Au plasmonic nanoarray showed an intense MEF effect due to the 3D space between Au nanostructures. Using this MEF-active platform, several nucleic acids, including bacterial genes and viral RNA, could be measured within 40 min and showed a high sensitivity that was 10-fold higher than the conventional RPA and PCR system. Li et al. measured the HIV-Ι trans-activation responsive (TAR) RNA-binding ligand, which is one of a potential biomarker of HIV, by aggregation-induced emission (AIE) with a metal-enhanced fluorescence (MEF) [78] (Figure 3d). For the induction of MEF, Fe_3_O_4_@Au@Ag@SiO_2_ nanoparticles were functionalized by TAR RNA, which was to specifically bind the tetraphenylethylene (TPE)-labeled TAT peptide. Once the target ligand was bound to the TAR RNA, the TPE was far away from the Fe_3_O_4_@Au@Ag@SiO_2_ nanoparticles, and the MEF effect was decreased. The LOD for each ligand could be obtained as 63 nM for neomycin B, 502 nM for chlorpromazine, and 36 nM for mitoxantrone, respectively. The authors claimed that this sensing system could provide a potential drug screening platform related to viral RNA-associated diseases. Shen et al. exhibited an ultrasensitive norovirus RNA quantification biosensor by integrating DNA-Ag nanocluster for the MEF effect [79]. The DNA-Ag nanocluster with a GCC-loop-structure could drastically change its fluorescence intensity if the target RNA was bound to the loop and formed a double-stranded structure, which made a secondary structure and induced turn on mode of the fluorescent signal of the Ag nanocluster. The detection limit of this RNA biosensor was 18 nM and the detection ranges were from 20 nM to 1.8 μM.

**Table 3:** Comparison of fluorescent-based plasmonic nanobiosensors for viral RNA detection.

Analytical Method	Feature	Target	Required Time	Detection Limit	Ref
Magnetofluorescence	Magnetic probes and HRP-terminated reporters generating HRP-catalyzed fluorescence readout	SARS-CoV-2 RNA	30 min	1000 copies/μL	[72]
Real-time plasmonic (fluorescent) RT-PCR	Multiplexed real-time plasmonic RT-PCR, with heating driven by IR LEDs and AuNRs	SARS-CoV-2 RNA	30 min	2.2–4.4 copies/μL	[73]
Plasmonic thermocycling and fluorescence detection	Control the temperature by the photothermal conversion of magneto-plasmonic nanoparticles	SARS-CoV-2 RNA	17 min	3.2 copies/μL	[74]
FRET	One-donor-two-acceptor system consisting of QD as a donor and BHQ-2, and AuNP as acceptors	RSV gRNA	40 min	-	[75]
MEF	Ai-Ni magnetoplasmonic nanorod with molecular beacon-fluorescent probe	miR-124	30 min	1 pM	[76]
MEF	3D Au plasmonic nanoarray composed of Au nanopillar and spherical AuNPs with RPA	SARS-CoV-2 RNA	40 min	10 copies/rxn	[77]
MEF	TAR RNA-immobilized surface of the Fe_3_O_4_@Au@Ag@SiO_2_ nanoparticles (NPs)	HIV-Ι TAR RNA-binding ligand	5 min	36 nM (mitoxantrone)	[78]
MEF	DNA-templated silver nanoclusters (DNA-AgNCs) with a GCC-loop-structure	Norovirus RNA	30 min	18 nM	[79]

### 4.2. Raman

**Figure 4:**
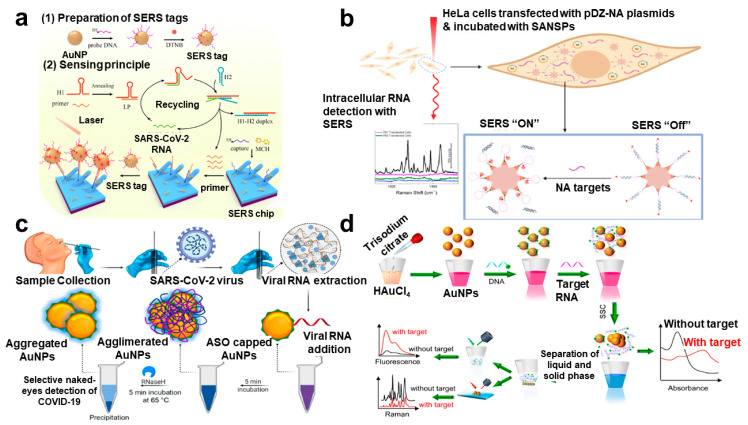
Viral RNA-target Raman and SERS-based biosensors using plasmonic nanomaterials: (**a**) Schematics of the point-of-care SERS biosensor using non-enzymatic signal amplification method. This figure is reproduced from [78] (© 2022 Elsevier B.V.); (**b**) Capture DNA-functionalized Au nanostar for the detection of intracellular viral mutation. This figure is reproduced from [79] (© 2022 American Chemical Society); (**c**) Plasmonic AuNPs-assisted colorimetric biosensor for detection of SARS-CoV-2 N gene RNA. This figure is reproduced from [81] (© 2022 American Chemical Society); (**d**) Colorimetric/SERS/fluorescence triple-mode plasmonic biosensor based on DNA-functionalized AuNPs. This figure is reproduced from [83] (© 2022 Elsevier B.V.).

As with the measurement of viral DNA, the Raman-based measurement has been consistently used for the diagnosis of viral RNA. In particular, the use of plasmonic nanomaterials in the process of converting the amount of RNA into a measurement signal can induce the amplifying of the existing Raman signal (SERS), so it can be effectively used for RNA detection, which is less stable than DNA and requires more sensitive measurement (Table 4). Nasir et al. exhibited the quantitative and qualitative analysis of hepatitis C virus (HCV) RNA from a blood sample by using the SERS-based method [80]. For the SERS phenomenon, AgNPs were applied as SERS substrates, and data analysis techniques such as partial least square regression (PLSR) and principal component analysis (PCA) were also integrated for the specific diagnosis. Because the SERS spectrum was complex and difficult to analyze, the PCA method should be helpful for the diagnosis of viral infection. After the PCA process, SERS data from patient samples showed a clear differentiation from its healthy samples (99% accuracy). Batool et al. showed the identification and differentiation of two comparable viral RNA diseases, HBV and HCV, using a PCR-integrated SERS analysis [81]. In this work, the authors also utilized the AgNP for the SERS effect and PCA analysis for the differentiation of SERS data from healthy, HBV, HCV, and negative samples. The partial least square discriminant analysis (PLS-DA) analysis facilitated checking the validity of the categorization of SERS data of HBV and HCV disease, and its specificity and sensitivity were provided as 96% and 94%, respectively. Zhang et al. developed a non-enzymatic signal amplification system for the ultrasensitive detection of the SARS-CoV-2 RNA [82] (Figure 4a). On the sensing substrate, Ag nanorods were functionalized for the enhancement of Raman signals, and capture DNA was immobilized for the hybridization of SERS probe-AgNPs. Due to the recycling reaction between target SARS-CoV-2 RNA, primer, and hairpin DNAs, AgNP can be attached to the chip surface in large numbers with enzyme-free amplification of the target RNA. Thus. The amount of viral RNA can be quantified sensitively by the intensity of the SERS signal, ranging from 10^2^ to 10^6^ copies/mL with 51.38 copies/mL of LOD. Dardir et al. developed the intracellular RNA biosensor for the monitoring of influenza RNA mutation using thiolated DNA hairpin and Raman dye-functionalized Au nanostar [83] (Figure 4b). DNA hairpin could be hybridized to the complementary sequence of viral RNA. If the viral RNA was bound to the complementary sequence, the SERS signal exhibited strong intensity. Using this mechanism, intracellular SERS signals in HeLa cells were examined with high selectivity for the hemagglutinin segment. The authors claimed that this sensing system successfully showed the applicability for the sensitive measurement and multiplexed detection of viral RNAs in individual cells.

### 4.3. LSPR-Based Analysis

Basically, plasmonic nanomaterials showed a particular optical property, and it often displayed the peak change of the absorbance wavelength or color change. This phenomenon originated from the LSPR of plasmonic nanomaterials. Qiu et al. developed the dual-functionalized plasmonic Au nanoislands for the improvement of sensitivity to detect SARS-CoV-2 RNA [84]. Plasmonic Au nanoisland could convert the optical energy to heat generation, which provided enhanced sensing stability, reliability, and sensitivity. In detail, the localized photothermal effect is capable of promoting the in situ hybridization temperature and enabling the accurate discrimination of two similar genes. In addition, Au nanoislands offered the LSPR-mediated biosensing substrate by measuring the change of absorbance property. Using a multigene mixture, selected SARS-CoV-2 RNA sequences were successfully detected as low as 0.22 pM. Ye et al. integrated the isothermal amplification method (LAMP) into the colorimetric assay through the Au–Ag alloy nanoshells [85]. Due to the condition that LAMP was feasible to amplify the target RNA, it could be one of the useful strategies to improve the sensitivity. Compared to the Au nanomaterial, Au–Ag alloy showed 4-times stronger extinction and 20-times more sensitive detection performance, as low as 10 copies per reaction within 70 min. Moitra et al. reported a very simple colorimetric biosensor for the SARS-CoV-2 viral RNA detection [86] (Figure 4c). The complementary sequence was modified on the surface of AuNPs, and SARS-CoV-2 viral RNA could induce the aggregation of AuNPs by the hybridization reaction. This aggregation could confirm by the color change of AuNPs and the red-shift of the absorbance peak. Using this change, measured viral RNA as low as 0.18 ng/μL. This visualized biosensor could be easily applied to on-site monitoring and other viral infection. Gao et al. exhibited a colorimetric/SERS/fluorescence triple-mode nanobiosensor based on plasmonic AuNPs for the effective diagnosis of SARS-CoV-2 infection [87] (Figure 4d). In this work, colloidal AuNPs were stabilized by the capture DNA, which was covered on the surface of the AuNPs and prevented salt-induced self-aggregation. Once the viral RNA complimentary hybridized to the capture DNA, AuNPs became an unstable state and easily aggregated each other. This caused the shift of the absorbance peaks (color change) of AuNPs, fluorescence intensity variation of supernatant, and reduction in SERS signal intensity from the capture DNA. Through the triple-mode sensing system, the accuracy and sensitivity (160 fM) of the nanobiosensor were improved with a reduction in the false negative of the detected signal within 40 min. As such, developed viral RNA biosensors using LSPR have the advantage of being able to diagnose with the naked eye and, at the same time, being able to measure a small amount of RNA in a short time.

**Table 4:** Comparison of SERS and LSPR-based nanobiosensors for viral RNA detection.

Analytical Method	Feature	Target	Required Time	Detection Limit	Ref
Raman	Silver nanoparticles (Ag NPs) as Raman substrates with multivariate data analysis technique, PCA and PLSR	HCV RNA	-	2.55 log IU/mL	[80]
SERS	PCR-integrated detection system, silver nanoparticles (Ag NPs) as SERS substrates with multivariate data analysis technique, PCA and PLS-DA	HBV and HCV RNA	-	-	[81]
SERS	SERS-active silver nanorods (AgNRs) sensing chips and a specially designed smart unlocking-mediated target recycling signal amplification	SARS-CoV-2 RNA	50 min	51.38 copies/mL	[82]
SERS	Gold nanostars functionalized with a Cy3-tagged beacon DNA for in vitro sensing	Hemagglutinin (HA) segment	-	-	[83]
LSPR	Two-dimensional gold nanoislands (AuNIs) functionalized with complementary DNA receptors	SARS-CoV-2 RNA	800 s	0.22 pM	[84]
LSPR	Gold and silver (Au–Ag) alloy nanoshells integrating LAMP method	SARS-CoV-2 RNA	75 min	10 copies/rxn	[85]
LSPR	Plasmonic AuNPs capped with suitably designed thiol-modified antisense oligonucleotides (ASOs) for colorimetric sensor	SARS-CoV-2 RNA	10 min	0.18 ng/μL	[86]
LSPR	Colorimetric/SERS/fluorescence triple-mode biosensor based on 17 nm-sized AuNPs	SARS-CoV-2 RNA	40 min	160 fM	[87]

## 5. Outlook and Conclusions

Recently, due to the epidemic of coronavirus, viral nucleic acid measurement technology has made a lot of progress in 3–4 years. In general, viral DNA and RNA were measured using a nucleic acid amplification method such as PCR, and biosensors using the plasmonic nanomaterials presented in this review were developed as one of the alternatives to compensate for the shortcomings of nucleic amplification-based methods. For more accurate and sensitive virus diagnosis, plasmonic biosensors are expected to have the following development directions in the future. First, it is necessary to synthesize plasmonic nanomaterials with better plasmonic effects than before. For example, in order to maximize the SERS effect, there is a method of changing the structure of nanomaterials or synthesizing and using several nanomaterials at the same time. The second is the fusion of data post-processing tasks such as artificial intelligence (AI) and machine learning (ML). Among the analysis methods described above, signals obtained through SERS are complex for humans to determine the infection or not. Because the interpretation of Raman peaks is important for the precise analysis of viral nucleic acids, if ML techniques are combined, more accurate and precise diagnostic results can be obtained. Third, it can be effectively integrated with the measurement method using CRISPR gene scissors, a nucleic acid diagnostic method that has been widely applied recently. Although research on diagnostic methods using CRISPR gene scissors is being actively conducted, research using plasmonic nanomaterials still needs more research for effective viral detection. If the advantages of CRISPR gene scissors and plasmonic nanomaterials are combined, better results can be obtained for measuring existing viral DNA and RNA.

In this review, we briefly discuss recent plasmonic biosensors that detect viral DNA and RNA. Plasmonic nanomaterials can serve to convert and amplify external light energy into various other optical readouts due to their unique surface plasmon properties. Using this, it was possible to rapidly and sensitively measure various viral DNA and RNA. In this summary, representative measurement methods include FRET and MEF using fluorescence, SERS amplifying Raman signals, and LSPR-based color-changing biosensors that can detect with the naked eye. In plasmonic biosensors for the detection of virus-derived nucleic acids, the advantages of each analytical method are different, but in common, the measurement sensitivity is improved, the required time is reduced, and a simpler measurement is possible. The development of such advanced plasmonic biosensors is expected to increase the survival rate of patients through accurate diagnosis of various nucleic acid-based fatal diseases as well as viral diseases in the future and enable early diagnosis and personalized treatment through gene analysis.

## Data Availability

Not applicable.
